# Understanding the future of dengue in Malaysia: Assessing knowledge, attitude, and homeowner practices in mitigating climate-driven risks

**DOI:** 10.12688/f1000research.157226.1

**Published:** 2024-11-12

**Authors:** Jo Ann Andoy-Galvan, Sapna Shridhar Patil, Yin How Wong, Priya Madhavan, Lei Hum Wee, Pei Pei Chong, Chung Yeng Looi, Imam Shaik, Anitha Ponnupillai, Ameya Ashok Hasamnis, Prabal Bhargava, Arasarethinam Mugilarasi, Eng Hwa Wong, Chai Hong Yeong, Weng Keong Yau, Mahalingam Dinesh, Kulankara Balan Venugopalan, Nor Asiah Muhamad, Nur Hasnah Ma’amor, Izzah Athirah Rosli, Fatin Norhasny Leman

**Affiliations:** 1 School of Medicine, Faculty of Health and Medical Sciences, Taylor's University, Subang Jaya, Selangor, 47500, Malaysia; 2 Digital Health and Medical Advancement Impact Lab, Taylor's University, Subang Jaya, Selangor, 47500, Malaysia; 3 School of Biosciences, Faculty of Health and Medical Sciences, Taylor's University, Subang Jaya, Selangor, 47500, Malaysia; 4 Sector for Evidence Based Healthcare, National Institute Health,Ministry of Health, Malaysia, Malaysia

**Keywords:** Keywords: dengue, knowledge, attitude, practices, PPR

## Abstract

**Introduction:**

Dengue fever poses a significant public health threat, particularly in tropical regions like Malaysia. The rising incidence of dengue outbreaks challenges healthcare systems and highlights the urgent need for effective preventive measures. Climate change, with rising temperatures and shifting rainfall patterns, is expected to worsen the dengue situation in the coming years.

**Methods:**

. This is a cross-sectional study conducted among adult residents of low-cost housing apartments in an urban poor community in Kuala Lumpur, Malaysia involving approximately 16,000 residents. A representative sample of 1,636 residents was calculated using the Krejcie and Morgan formula, and stratified random sampling was used to ensure proportional representation across the various floors of each PPR community apartment block. Data were collected using a structured questionnaire adapted from a validated Malay version. The questionnaire assessed respondents’ knowledge, attitudes, and practices (KAP) related to dengue prevention, categorising KAP scores as “Good” (≥80%) or “Poor” (<80%). Descriptive statistics summarized population characteristics and KAP scores, while logistic regression identified predictors of KAP levels, with significance set at p ≤ 0.05.

**Results:**

In this community, 76.7% of participants exhibited poor knowledge and 83.1% had a negative attitude towards dengue, despite 66.7% demonstrating good preventive practices. The PPR location significantly predicts dengue knowledge, attitudes, and preventive practices, with p-values of less than 0.001 for all domains. Marital status also predicts dengue knowledge (p = 0.007) and preventive practices (p = 0.023), while prior infection with dengue is a predictor of preventive practices (p = 0.047).

**Conclusion:**

Despite the community’s good dengue prevention practices, likely influenced by environmental expectations, there remains a critical need for education to sustain and strengthen these efforts, as climate change continues to worsen in the coming years. It is crucial to help residents grasp the relevance of these practices, so they can apply them more effectively as climate-driven risks intensify. Targeted interventions should de designed differently for each of the four PPR communities as their levels of knowledge, attitude and practices vary significantly, taking into account independent factors like marital status and prior dengue infection, which shape preventive behaviors.

## Introduction

### Climate change and Dengue

Climate change has emerged as a pressing global issue, with extensive consequences on environmental, social, and public health aspects. The increasing intensity of extreme weather conditions, rising temperatures, and shifting precipitation patterns are exacerbating existing challenges leading to a range of adverse health outcomes, including the spread of vector-borne diseases. These diseases are particularly susceptible to the impacts of climate change due to alterations in vector habitats and disease transmission dynamics. The modified environment creates favourable conditions for vector proliferation and disease transmission, leading to increased incidence and geographic spread of these diseases ( Vieira et al., 2024).

Malaria and Dengue fever are among the most prevalent vector-borne diseases worldwide, with an estimated incidence of 249 million cases of Malaria ( WHO, 2023) and 100-400 million cases of Dengue in 2022 ( WHO, 2024). Warmer temperatures are expanding the geographic range of mosquitoes responsible for transmitting malaria (Anopheles) and dengue (Aedes). Cases are now being reported more frequently in countries with cooler climates, where they were rarely reported previously ( Lenharo, 2023). Heavy rainfall and flooding create conducive environments for mosquito breeding. Stagnant water pools formed during floods serve as their ideal breeding.

### Dengue incidence

Malaysia has entered the elimination phase of malaria control, but Dengue fever remains a growing problem ( Sa-Ngamuang et al., 2023; Md Taib & Atil, 2023). Dengue fever is a prominent public health challenge which often affects the tropics. It is brought upon by the flavivirus transmitted by the Aedes aegypti mosquito. In Malaysia, despite various control measures, the incidence of dengue continues to rise, ( Chem et al., 2024), with a 235.6% increase in cases reported between December 31, 2022, and June 10, 2024 ( Tan & Gerard, 2024). A retrospective study conducted by Bujang et al. aimed to investigate the trend in dengue cases in Malaysia revealed a staggering 1561.3% increase from 1995 to 2014. The study also reported an incidence rate of 161.5 per 100,000 population in 2010 and is projected to rise to 940.0 per 100,000 population by 2040 ( Bujang et al., 2017).

**Figure 1.**
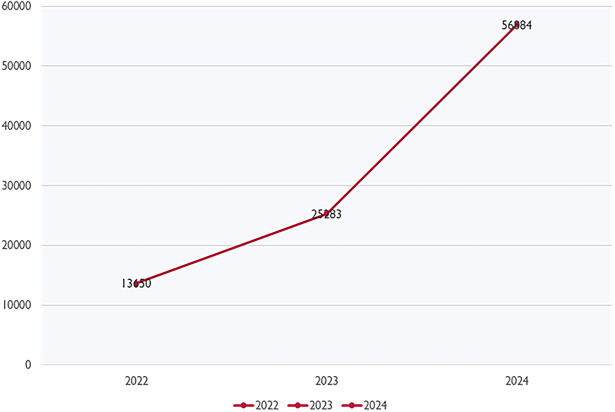
Dengue cases from 2022 to 2024 (Jan to May). Source from National Crisis Preparedness and Response Centre (CPRC), Ministry of Health Malaysia (MOH).

As of May 10, 2024, a total of 56,884 dengue cases have been reported, covering the period from December 31 of the previous year up to the present day. The daily average for new cases stands at 334 (see Table 1). From 2022 to 2024 (accumulated cases from January to May), there was a substantial increase of 315.23% in dengue cases ( Wan-Fei et al., 2023) (See Figure 1). Similarly, from 2023 to 2024 (accumulated cases from January to May), there was a notable increase of 124.20% in dengue cases ( Ministry of Health Malaysia 2023).

**Table 1.** Daily cases and accumulated cases in the different states of Malaysia 2024 (as of May 10, 2024).

State	May 10, 2024 Dengue cases	Dec 31 - May 10, 2024 Dengue cases
Johor	30	6270
Kedah	16	1815
Kelantan	6	864
Melaka	9	814
Negeri Sembilan	9	2969
Pahang	1	639
Pulau pinang	12	2019
Perak	22	3353
Perlis	0	77
Selangor	161	29678
Terengganu	0	112
Sabah	11	1829
Sarawak	13	897
Wilayah Persekutuan	44	5537
Wilayah Persekutuan Labuan	0	11
Malaysia	334	56884

* Source from iDengue_Versi 3.0. (May 10, 2024). Retrieved from https://idengue.mysa.gov.my/.

### Current established Dengue prevention and control measures by the Ministry of Health, Malaysia

States like Selangor, with densely populated urban areas, report the highest incidence rates, exacerbated by poor housing conditions that contribute to mosquito proliferation. The steep rise in dengue cases can be attributed not only to environmental factors but also to the limitations of current prevention measures. PKD in Selangor is grappling with a substantial burden as the region contends with a surge in dengue cases. As of May 12, 2024, 29,678 cases have been reported, accompanied by the identification of 124 hotspot areas ( idengue.mysa.gov.my, 2024).

PKD employs several commendable initiatives in managing the dengue situation in their locality ( Rusli et al., 2022), encompassing early detection facilitated by the integrated reporting system through e-notifikasi, strict enforcement of reporting protocols, and targeted destruction of breeding sites. Doctors are mandated to report dengue cases within 24 hours under the “Act 342 Prevention and Control of Infectious Diseases Act 1988,” with non-compliance incurring a fine of RM 1000. Once a case is reported, the PKD registers reported cases to assess outbreak potential, defined as two cases in a locality or one case residing 200 meters from the index case or first case within 14 days.

In response to outbreaks, the PKD conducts fogging and search-and-destroy operations. Health officers will conduct fogging within a 400-meter radius from the reported outbreak’s address, repeating these cycles every 5-7 days which continues for 2 weeks after the outbreak. They also identify breeding sites, with RM 500 penalties for house owners where breeding sites were identified. Larviciding activities are initiated and repeated every 3-5 days until 2 weeks after the resolution of the outbreak.

**Figure 2.**
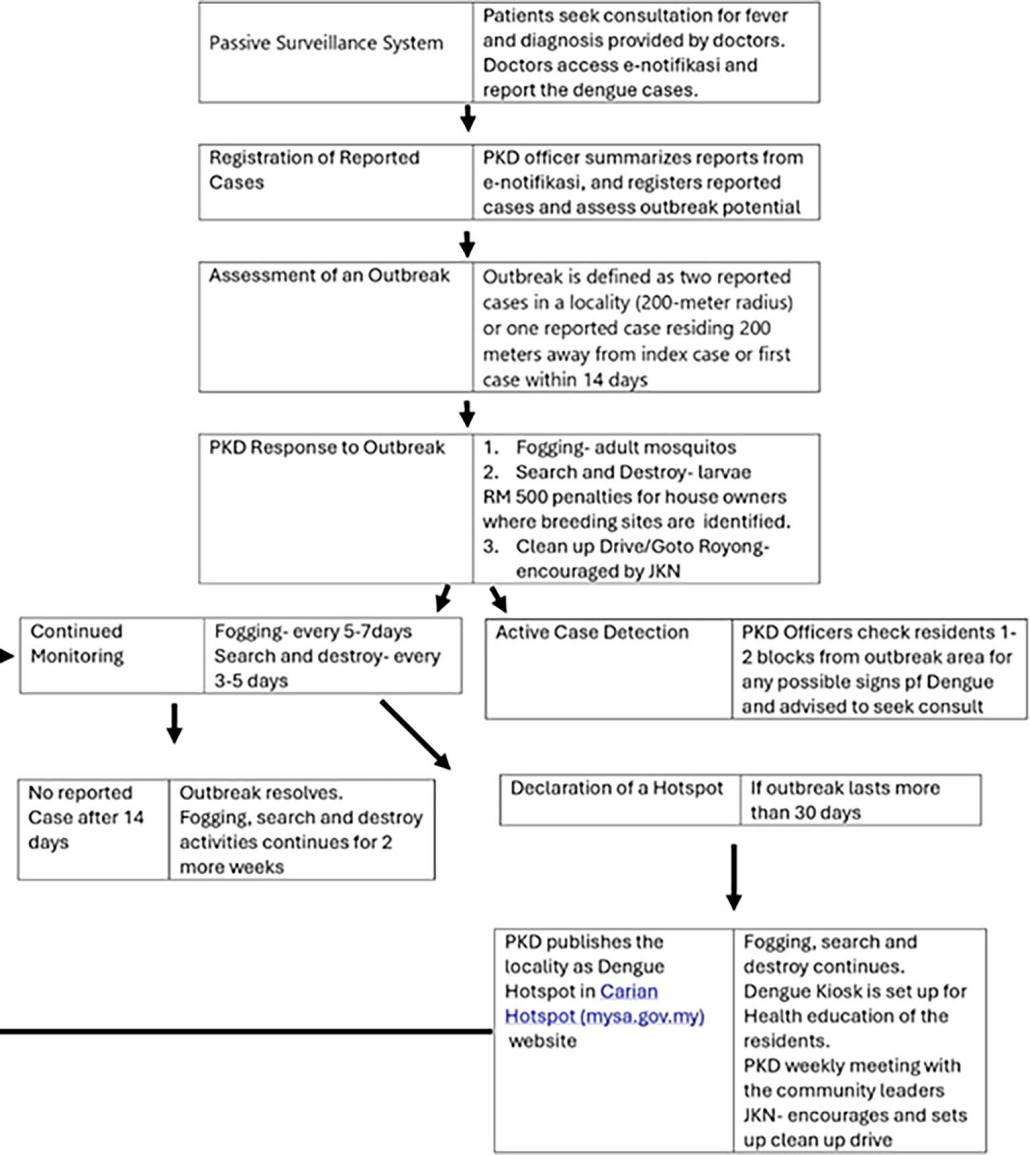
Dengue Outbreak Management, PKD Gombak Malaysia. PKD = Pejabat Kesihatan Daerah (District Health Office), E-notifikasi = online notification system used for reporting certain communicable diseases, JKN = Jabatan Kesihatan Negeri (State Health Department). Source: Pelan Strategik Pencegahan Dan Kawalan Denggi Kebangsaan 2022-2026.

However, if these areas continue to have reported cases after 30 days, they will be considered as Dengue Hotspots and will be published on the “idengue” website. PKD will work closely with these communities, by meeting them weekly for weekly presentation of cases and education. The State Health Department or the Jabatan Kesihatan Negeri (JKN) will also encourage the community to do clean-up drives or the “Gotong Royong” in Malay ( Figure 2).

Environmental factors are key determinants in the proliferation of dengue vectors and the subsequent outbreak of the disease. Poor waste management practices, including irregular garbage collection and improper disposal of waste, contribute significantly to the breeding of mosquitoes. In urban areas, where population density is high, and infrastructure may be inadequate, stagnant water in discarded containers, blocked drains, and neglected water storage vessels become ideal breeding sites for Aedes mosquitoes.

Collaborative efforts between government agencies, local authorities, community organisations, and residents are essential to creating sustainable solutions that mitigate the environmental factors contributing to dengue outbreaks.

The strain on resources is palpable, with the PKD stretched thin in its efforts to combat the escalating dengue crisis. Hotspot areas, identified as focal points of dengue transmission, continue to report cases over extended periods, indicating persistent challenges in eradication efforts. Compounding these challenges is the overwhelming caseload experienced during massive outbreaks, leading to lapses in essential activities like active case detection and comprehensive environmental risk assessments ( Tee et al., 2019).

This scenario underscores the urgent need for bolstered resources, strategic interventions, and enhanced collaboration between stakeholders to effectively address the dengue epidemic in Selangor. Without concerted efforts to strengthen the capacity of the PKD and implement targeted measures to curb dengue transmission, the region may continue to face prolonged and severe public health challenges associated with the disease.

The government has invested significant resources in vector control efforts and imposed fines for failing to eliminate mosquito breeding grounds. However, many of these breeding sites are located within private homes, where government interventions are limited. The real issue lies deeper: at the heart of the problem are the residents’ practices regarding dengue prevention. Effective prevention starts with homeowners taking responsibility for eliminating potential breeding grounds on their properties. But for these practices to be adopted, residents must first recognize their importance. This requires not only knowledge about dengue prevention but also a shift in attitude. Residents must perceive their susceptibility to infection and understand the severity of the disease and its impact on their families and daily lives. Only when they fully grasp these risks can they embrace preventive measures and protect their communities from dengue.

### Gaps in current Dengue control & prevention practice

A study by Zaki et al. involving 847 residents in a Selangor district, reported that the majority have a good understanding of dengue fever (97.1%) and its correlation with climate factors (90.6%). A significant percentage expressed willingness to contribute to reducing dengue cases in their locality (91.5%). However, over half of the respondents (64%) do not regularly inspect potential hotspots in their area, and not everyone is eager to participate in cleanup initiatives ( Zaki et al., 2019). Correct understanding or knowledge does not necessarily result in the adoption of correct attitudes and practices for dengue prevention A scoping review done by Rahman et al., on identifying the barriers to knowledge, attitude and practices of dengue prevention in the community involving 349 articles from 2010-2018 suggests that factors interfering with dengue prevention practices are divided into two, namely:

1. the internal factors that encompass attitudes and perceptions of dengue prevention
2. and the environmental factors such as weather, drainage systems, buildings designed with unreachable rain gutters and poor drainage and piping systems.

**Figure 3.**
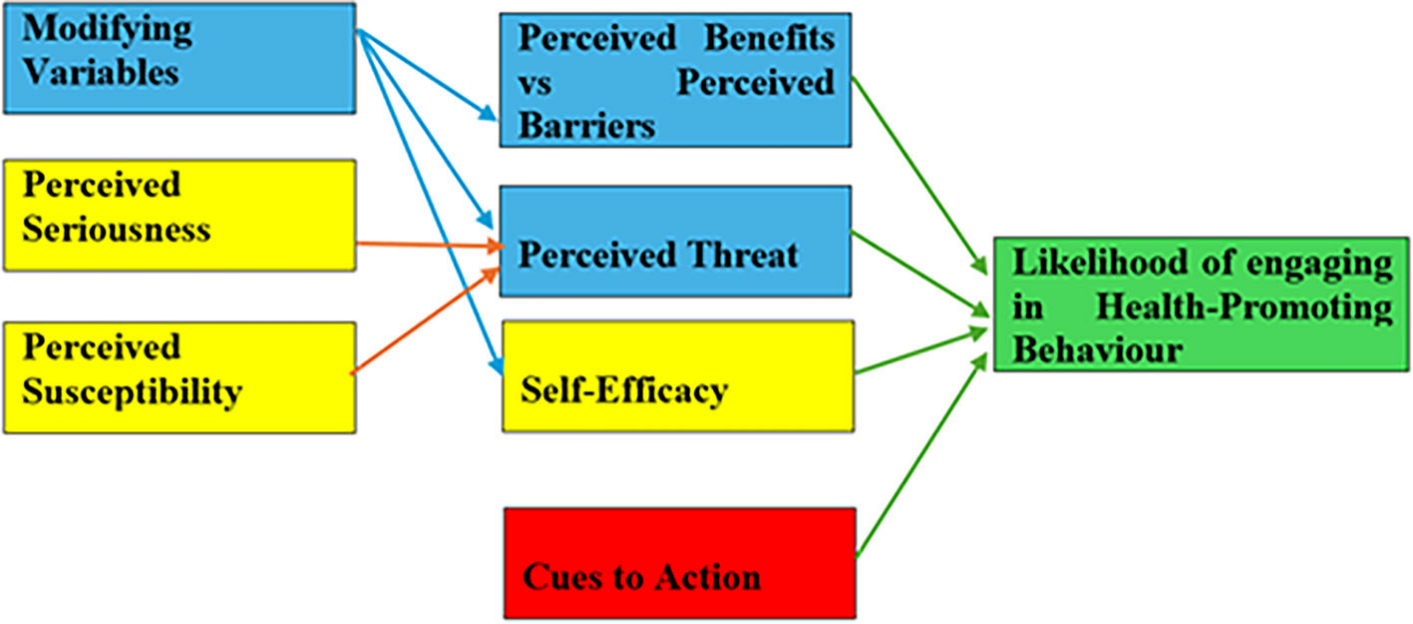
Health belief model.

To mitigate the ongoing dengue problem, the government must prioritize densely populated areas like Selangor and Kuala Lumpur, focusing not only on these environmental factors but also on the internal factors that drive residents’ prevention practices. An effective strategy for combating dengue requires addressing residents’ understanding of their vulnerability to infection and the severity of its potential impact. The Health Belief Model (HBM), a widely used framework in public health, provides valuable insights into how individuals’ perceptions influence their health-related behaviors. It explains that people are more likely to adopt preventive measures when they perceive themselves to be at risk (perceived susceptibility) and understand the serious consequences of the disease (perceived severity). In the context of dengue prevention, if residents recognize their vulnerability to infection and believe that the impact of dengue can be severe, they are more likely to take appropriate actions, such as eliminating mosquito breeding sites and using protective measures. According to Glanz et al. (2008), the HBM underscores the importance of addressing both external conditions and internal beliefs to effectively promote behavior change and eventually for this particular case, reduce the spread of dengue ( Figure 3).

Residents of low-income communities may underestimate their susceptibility to the virus. Environmental factors such as overcrowded living conditions and poor waste management in these areas create ideal breeding grounds for mosquitoes. They are the ones vulnerable to the financial burden of contracting dengue. A single day of lost income due to illness can have dire economic consequences for households already struggling to make ends meet.

Dengue fever is often referred to as a disease prevalent in poor areas, and closely linked with impoverished populations. The World Health Organization categorises dengue fever as one of the neglected tropical diseases (NTDs), which are indicative of poverty, and recommends interventions targeted at populations in poverty-stricken and marginalised communities to address dengue ( Mulligan et al., 2015).

In Malaysia, where dengue remains a significant public health threat, there is a notable scarcity of data on the national understanding of dengue infection, as well as on public attitudes and preventive practices. Therefore, this study aimed to assess the knowledge, attitudes, and preventive practices of a low-income community consisting of 16,000 residents in a densely populated area of Kuala Lumpur. The results of this study are expected to contribute to the development of evidence-based community health intervention programs that specifically target homeowners’ preventive practices. These findings will enhance our understanding of the factors influencing knowledge, attitudes, and practices among low-income, community-dwelling adults and provide public health authorities with crucial insights for prioritizing actions in similar settings across Malaysia.

## Methods

### Study design, population and sample size

***Study design and setting***

A community-based, cross-sectional study was conducted over 4 weeks in July 2024 among adult residents of four low-cost housing apartments under the People’s Housing Programme (Program Perumahan Rakyat or PPR) in the federal constituency in Kuala Lumpur, Malaysia. In 1998, the National Housing Department of the Malaysian Ministry of Housing and Local Government (Jabatan Perumahan Negara) developed the PPR to provide affordable housing for Malaysians of low socioeconomic status with a monthly household income below RM 3,000 ( Goh et al., 2011).

The four participant PPRs in this study were PPR Sri Pantai, PPR Pantai Ria, PPR Kampung Limau and PPR Seri Cempaka. These PPRs were chosen for their proximity to the Klang River, which creates significant challenges for vector control due to stagnant water that serves as breeding grounds for Aedes mosquitoes. Our aim is to assess the community’s understanding of these risks to develop an effective health intervention for this vulnerable area.

**Figure 4.**
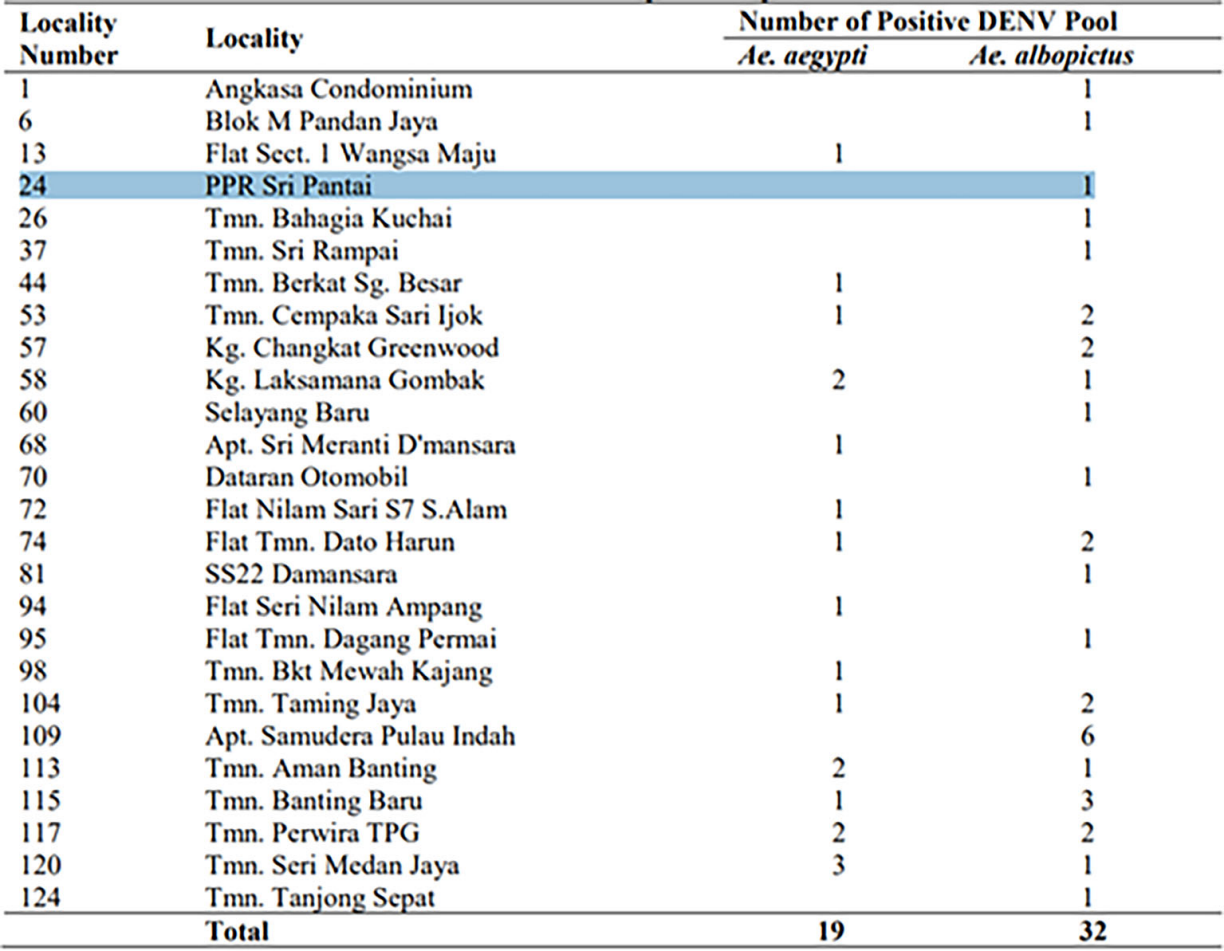
Distribution of 51 DENV-positive pools collected in 26 localities. Source: 2021 study by the National Institute of Health in collaboration with the University of Malaya ( Wan et al., 2021).

Dengue has been confirmed in this community, as shown by the DENV-positive mosquito pools recorded in the table below based on a 2021 study by the National Institute of Health in collaboration with the University of Malaya ( Wan et al., 2021). We have specifically chosen this community for the survey because such areas, especially near water bodies, are prone to recurrent outbreaks, highlighting the necessity of continuous monitoring and targeted interventions ( Figure 4).

### Sampling frame, design and sample size

Each of these PPRs comprises multistorey apartments that are labelled as blocks. PPR Sri Pantai has two 21-storey blocks namely, Block 100 and 102 with 20 units on each floor. Each of the four blocks A, B, C and D of PPR Pantai Ria has 17 floors, with a total of 20 units per floor. Block 96 and Block 94 of the PPR Kampung Limau are 17-storeyed with 20 units per floor. The Seri Cempaka PPR consists of two 17-storey blocks, ‘E’ and ‘F’. To minimize sampling bias, a structured design was used, with the sampling frame drawn from four PPR areas. Stratified random sampling ensured proportional representation from all floors of each apartment block. Data collection included weekends to ensure broader resident participation. This approach helped capture a diverse and representative sample, reducing the risk of bias related to availability or uneven representation.

The sample size is calculated using the Krejcie and Morgan sample size calculation method ( Krejcie and Morgan, 1970) to ensure it is representative of the entire population.

Sample size calculation: The formula for the Krejcie and Morgan sample size calculation is as follows:

$$n=\frac{X^{2}.N.P\;\left(1-P\right)}{\left(N-1\right).d^{2}+X^{2}.P\;\left(1-P\right)}$$

• s = required sample size
• X ^2^ = the table value of chi-square for 1 degree of freedom at the desired confidence level (3.841 for 95% confidence level)
• N = the population size
• P = the population proportion (assumed to be 0.5 for maximum sample size)
• d = the degree of accuracy expressed as a proportion (0.05 for 95% confidence level)

Using this formula, the following sample sizes have been calculated for the populations. The estimated sample size for each of the PPRs based on the total population is shown in Table 2.

**Table 2.** Sample size calculation for each participating PPR.

Name of PPR	Total population	Minimum sample size	Blocks	Floors
PPR Sri Pantai	4,000	350	2	21
PPR Pantai Ria	6000	361	4	17
PPR Kampung Limau	2500	333	2	17
PPR Seri Cempaka	2800	300	2	17

PPR = Program Perumahan Rakyat (People’s Housing Project).

### Questionnaire

The structured questionnaire was adapted from the validated Malay version developed by Mac Guad et al. (2021). Permission to use the questionnaire and scoring system was obtained from the authors. The questionnaire was divided into four sections:

1. Socio-demographic Information: This section gathered data on age, gender, ethnicity, marital status, education level, occupation, household income, and family size.
2. Knowledge (Part A): Nine questions assessing respondents’ knowledge of dengue transmission, symptoms, and prevention.
3. Attitudes (Part B): Eight questions gauging attitudes toward dengue prevention measures and public health policies.
4. Practices (Part C): Eleven questions focusing on the respondents’ actual behaviors in preventing dengue, such as eliminating mosquito breeding sites and using preventive measures.

The KAP scores were calculated, with high KAP defined as achieving 80% or more of the maximum possible score, and low KAP defined as scores below this threshold ( Elia-Amira et al., 2023).

### Categorisation of knowledge, attitude, and practices

Respondents’ dengue knowledge, attitude, and practices (KAP) were categorised as “Good” or “Poor” based on predefined scoring criteria ( Table 4).

**Table 4.** Dengue Knowledge, Attitudes & Practices among Residents of PPR Lembah Pantai (N=1,636).

Level		f	%
Knowledge	Good	381	23.3
	Poor	1255	76.7
Attitude	Good	1359	16.9
	Poor	277	83.1
Practice	Good	1091	66.7
	Poor	545	33.3

PPR= Program Perumahan Rakyat (People’s Housing Project).

Below are the criteria used for each domain:

1. Knowledge: Respondents were assessed on their knowledge about dengue through a total of 25 questions, which included sub-questions under 9 main topics. To be categorized as having “good” knowledge, respondents needed to answer at least 20 questions correctly (80% or more). Those who scored below 20 were classified as having “poor” knowledge.
2. Attitude: The attitude section contained 8 questions. A score of 6 or more correct answers indicated a good attitude toward dengue prevention, while scores below 6 were categorized as poor attitude.
3. Practices: There were 11 questions assessing preventive practices against dengue. Respondents who correctly answered 9 or more questions were classified as practicing good preventive measures, while those scoring below 9 were considered to practice poor dengue prevention.

### Data analysis

Data analysis was conducted using IBM-SPSS Statistics version 25. Descriptive statistics, including frequencies and percentages, were used to describe the population characteristics and summarize participants’ knowledge, attitudes, and practices (KAP) related to dengue prevention. Logistic regression was used to identify potential predictors among the sociodemographic variables. Variables with a significance level of p ≤ 0.25 in univariate analysis were further analyzed using multiple logistic regression to determine independent predictors of high or low KAP levels. A p-value of ≤ 0.05 was considered statistically significant.

### Ethical considerations

Ethical approval for this study was obtained from the Institutional Review Board (IRB) of Taylor’s University Center for Research Management, with approval number HEC 2024/179, granted on June 20, 2024. The study adhered to the ethical principles outlined in the Declaration of Helsinki. Written informed consent was obtained electronically via a Google form. Participants were fully informed about the study’s purpose, and their rights to privacy and confidentiality were upheld. All collected data were anonymized to protect participants’ identities, and they were notified of their right to withdraw from the study at any time without any repercussions.

## Results

### A. Sociodemographic characteristics

The study collected 1,636 responses, with the majority being females (56.8) and Malays (78.3%). Most respondents were married (70.9%) and had secondary level education (64.4%). Of the total population, 47.35% are not working, with almost half being housewives at 26.3%, while the remainder consists of those who are either retired at 10.3% or unemployed at 10.7%, A significant portion earned less than RM2500 monthly (62.4%) (Table 3) I (extended data).

### B. Knowledge, attitude and prevention practices

In this community, 76.7% of participants exhibited poor knowledge and 83.1% had a negative attitude towards dengue, despite 66.7% demonstrating good prevention practices ( Table 4).

***Knowledge***

Most residents (77.7%) had limited knowledge about dengue. Most of the participants had correctly identified the symptoms, i.e. fever (88.9%) and nausea (68.2%) as symptoms, but wrongly identified cough (47.1%) and swollen glands (32%). Majority of the residents correctly identified mosquito behavior, but 43.3% incorrectly believed there’s a specific treatment for dengue. Most recognized the importance of rest (82.6%) and hydration (90.2%) for recovery, and 90.8% were aware of fines for mosquito larvae on their property (Table 5) (extended data).

***Attitude***

In terms of attitudes towards dengue prevention, a significant portion of residents (62.3) believe that killing the mosquitoes is the only way to prevent infection, with 66.8 percent trusts in fogging as a way of control. And the majority agrees (74.6%) that fines help in the control and believes that dengue is preventable (60.2%) ( Table 6).

**Table 6.** Dengue attitudes among residents of PPR Lembah Pantai (N=1,636).

Dengue attitude questions	Disagree		Neutral		Agree	
	f	(%)	f	(%)	f	(%)
A1: Dengue is a type of disease that cannot be prevented	985	60.2	192	11.7	459	28.1
A2: Disposal of mosquito larvae is a waste of time and troublesome	1167	71.3	175	10.7	294	18
A3: Killing mosquitoes carrying dengue is the only way to control or prevent infection	392	24	224	13.7	1020	62.3
A4: Eradicating mosquito breeding grounds is the responsibility of health professionals and volunteers only	909	55.6	193	11.8	534	32.6
A5: Supervised control and monitoring of suspected mosquito breeding grounds should be undertaken at least once a year	365	22.3	206	12.6	1065	65.1
A6: 'Fogging' can prevent mosquito breeding completely	279	17.1	264	16.1	1093	66.8
A7: Fines can help towards control of dengue	209	12.8	206	12.6	1221	74.6
A8: Health professionals are not required to inspect residential properties	823	50.3	287	17.5	526	32.2

PPR = Program Perumahan Rakyat (People’s Housing Project).

***Practice***

Most residents engaged in good dengue prevention practices. Out of the 11 dengue practices, majority about 75% of the respondents answered yes to all the questions, except with the Abate use (54.4%) ( Table 7).

**Table 7.** Dengue practices among Residents of PPR Lembah Pantai (N= 1,636).

Dengue practice questions	No		Yes		Not sure	
	f	(%)	f	(%)	f	(%)
P1: Are containers of water/wells in your residence covered?	278	17	1302	79.6	56	3.4
P2: Do you close water containers/wells once you have finished using them?	147	9	1445	88.3	44	2.7
P3: Do you always check the condition of stored water in containers/wells if you do not use them for more than five days?	207	12.7	1364	83.4	65	4
P4: Do you put an Abate (insecticide) in the reservoir in your residence?	677	41.4	890	54.4	69	4.2
P5: Have you ever checked for mosquito larvae in a discarded flower vase?	405	24.8	1150	70.3	81	5
P6: If there is stagnant water in a flower vase, would you throw it away?	204	12.5	1389	84.9	43	2.6
P7: Have you ever checked your residence and property for a container/place that could provide a mosquito breeding site?	246	15	1329	81.2	61	3.7
P8: Do you remove containers that can collect water and allow it to stagnate?	252	15.4	1320	80.7	64	3.9
P9: Do you check and clean the gutters of your residence each rainy season?	375	22.9	1207	73.8	54	3.3
P10: Do you consider eradicating Aedes mosquitoes is a shared responsibility?	69	4.2	1523	93.1	44	2.7
P11: Would you allow the authorities to conduct dengue prevention activities at your residence?	91	5.6	1491	91.1	54	3.3

PPR= Program Perumahan Rakyat (People’s Housing Project).

### C. Predictors of KAP

***Knowledge predictors***

To identify predictors of dengue knowledge, attitudes, and preventive practices, we initially conducted a univariate logistic regression analysis. Variables that demonstrated a significance level of 0.25 were subsequently included in a multivariate analysis. Among all tested variables, only PPR (Public Housing Program), marital status, and education level emerged as independent predictors of dengue knowledge.

The analysis revealed that residents from PPR Pantai Ria were 1.7 times more likely to have a higher level of dengue knowledge compared to those from PPR Sri Pantai (p = 0.004). Similarly, residents from PPR Sri Cempaka and PPR Kampung Limau demonstrated 2.8 times (<p = 0.001). and 3.2 times (p ≤0.001) higher odds, respectively, of possessing greater dengue knowledge relative to those from PPR Sri Pantai.

In terms of marital status, married residents were 3 times more likely to have good dengue knowledge compared to widowed or divorced residents (p = 0.007). This suggests that marital status, alongside location, plays a critical role in influencing residents’ awareness and understanding of dengue prevention and control.

Educational attainment is also an important predictor of dengue knowledge (p value≤0.002), even though no individual education level (primary, secondary, tertiary) showed a statistically significant association (Table 8) (extended data).

***Attitude predictors***

All variables were initially tested using univariate logistic regression to assess predictors of dengue-related attitudes, and those with a significance level of 0.25 were included in a multivariate analysis. Among the variables examined, only the PPR community was identified as a significant predictor of dengue attitude.

Residents of PPR Pantai Ria were found to be 2.7 times more likely to demonstrate a positive attitude towards dengue prevention compared to those from PPR Sri Pantai (p value≤0.001), while PPR Kampung Limau were 2 times more likely to exhibit favorable attitudes towards dengue prevention as compared to residents of PPR Sri Pantai (p value ≤0.001) (Table 9) (Extended data).

***Preventive Practices predictors***

To identify predictors of dengue preventive practices, we performed a univariate logistic regression analysis on all variables, followed by multivariate analysis for those with a significance level of 0.25. Our findings revealed three significant predictors of good preventive practices: PPR community, marital status, and previous infection with dengue.

In terms of the PPR communities, residents of PPR Pantai Ria were 2.7 times more likely to engage in dengue preventive practices compared to those from PPR Sri Pantai. Similarly, residents from PPR Sri Cempaka and PPR Kampung Limau had odds ratios of 1.9 (p < 0.001) and 1.7 (p < 0.001), respectively, for practicing dengue prevention relative to PPR Sri Pantai.

Marital status was another significant predictor. Both single and married individuals had higher odds of practicing dengue prevention compared to widowed or divorced residents, with odds ratios of 1.9 for singles (p = 0.015) and married individuals (p = 0.007), respectively.

Interestingly, prior infection with dengue was also a significant predictor. Those who had previously been infected with the virus were 1.5 times more likely to engage in preventive practices compared to those who had never experienced dengue (p = 0.047). This suggests that personal experience with the disease can enhance adherence to preventive measures (Table 10) (extended data).

## Discussion

The overall findings of this survey underscore the urgent need for the community to recognize the significance of their preventive practices. Despite demonstrating good preventive measures, approximately three-fourths of the population exhibit poor knowledge about dengue, raising concerns for the future. While these practices are commendable, sustaining them is vital, particularly considering escalating dengue threats exacerbated by climate change in the coming years.

Interestingly, Malaysia ranks first in implementing preventive measures according to a recent global systematic review and meta-analysis of knowledge, attitudes, and practices regarding dengue fever among the general population ( Jahromi et al., 2024; Aung et al., 2016; Yussof et al., 2017). This achievement may be attributed to the high incidence of dengue in the country, prompting residents to receive education on appropriate preventive actions.

However, a discrepancy exists between attitudes and knowledge in this particular community. Typically, one would expect that individuals with strong knowledge and understanding of dengue would translate this knowledge into effective practices. Conversely, those with poor knowledge are likely to neglect preventive measures ( Naqvi et al., 2024; Selvarajoo et al., 2020). In this specific community, however, the situation appears different. Many residents engage in preventive behaviours not necessarily because they fully understand the disease or possess a positive attitude toward dengue prevention, but rather probably out of a sense of obligation or expectation given that the country is experiencing high incidence of dengue.

To enhance community understanding and sustain such practices, a targeted community-based health education program is recommended. This program should focus on bridging the knowledge gap by providing interactive workshops, accessible educational materials, and practical demonstrations. Engaging local leaders and healthcare providers can foster a supportive environment that encourages active participation.

In the analytical analysis, the findings indicate that the PPR community is a significant independent predictor across the domains of dengue knowledge, attitude and preventive practices, with residents from various neighbourhoods such as Pantai Ria, Sri Cempaka, and Kampung Limau exhibiting different levels in comparison with Sri Pantai. These discrepancies are likely influenced by variations in access to health education, community engagement initiatives, and available resources.

A meta-analysis by O’Mara-Eves et al. (2015) emphasizes that localized interventions can substantially improve community health outcomes by raising awareness and mobilizing local resources. Engaging communities through targeted initiatives has shown positive effects on diverse health outcomes. Therefore, it is imperative for community leaders to play an active role in fostering collaborative efforts, such as organizing clean up drives or informative sessions, and designing health education campaigns that resonate with the specific needs and preferences of their communities. By facilitating inclusive activities that engage all residents, these community-driven initiatives can enhance health-related attitudes and promote proactive measures for dengue prevention, thereby contributing to improved public health outcomes.

Marital status emerged as a significant predictor of both dengue knowledge and preventive practices, with married individuals showing higher levels of engagement in dengue prevention, likely due to their increased responsibilities toward family health, particularly in households with children. Family dynamics and social support networks positively influence health-related behaviours ( Tunçgenç et al., 2023). Strategies must address the knowledge gaps and prevention behaviours among singles, widows, and divorced individuals, as these groups may not experience the same social pressures or support systems that encourage active participation in health initiatives. Tailored campaigns should be developed to help these individuals understand their personal risk and the importance of dengue prevention. Community leaders can foster a “family-like” environment, promoting mutual trust and commitment to collective well-being. By building a sense of community responsibility, individuals who may otherwise feel disconnected from health initiatives can be encouraged to participate.

Lastly, previous infection with dengue was identified as a significant predictor of preventive practices. Individuals who have experienced dengue firsthand are likely more aware of its impact and the importance of prevention. Personal experience with health issues often drives individuals to adopt more proactive health behaviours ( Short and Mollborn, 2015). Prior to infection, many may not have engaged in preventive practices; however, the experience of contracting dengue and the associated physical, emotional, and financial hardships appear to motivate behaviour change. Once individuals recognize their susceptibility to the disease and face consequences such as loss of income or the burden on their families, they become more likely to adopt preventive measures.

Public health education should leverage these insights by focusing on the tangible risks and consequences of dengue, particularly for those in vulnerable communities. Educational campaigns should highlight the specific conditions that increase susceptibility to the disease, such as overcrowded living environments, proximity to stagnant water, and inadequate waste disposal. It’s essential to make the connection between these environmental factors and their practical implications, like loss of income, disruption of family life, and the severe health risks, including fatal outcomes, associated with dengue. By personalizing these risks and emphasizing the potential for severe illness, public health messaging can resonate more deeply and encourage sustained community engagement in dengue prevention efforts.

## Conclusion

The findings of this survey underscore the need for the community to deepen their understanding of the relevance of their preventive practices. Although preventive behaviours are generally strong, three-fourths of the population exhibit poor knowledge of dengue, which may hinder the sustainability of these practices in the long term, particularly as the threat of dengue is exacerbated by climate change. The PPR community has been identified as a significant independent predictor of dengue-related knowledge, attitudes, and preventive practices. Marital status is an independent predictor of both dengue knowledge and preventive practices, and a history of prior dengue infection is a predictor of increased engagement in preventive practices.

### Ethical considerations

Ethical approval for this study was obtained from the Institutional Review Board (IRB) of Taylor’s University Center for Research Management, with approval number HEC 2024/179, granted on June 20, 2024. The study adhered to the ethical principles outlined in the Declaration of Helsinki. Written informed consent was obtained electronically via a Google form, ensuring voluntary participation. Participants were fully informed about the study’s purpose, and their rights to privacy and confidentiality were upheld. All collected data were anonymized to protect participants’ identities, and they were notified of their right to withdraw from the study at any time without any repercussions.

## Data Availability

Dataverse: “Understanding the future of Dengue in Malaysia: Assessing Knowledge, Attitude, and Homeowner Practices in Mitigating Climate-Driven Risks”, https://doi.org/10.7910/DVN/9VNEET ( Galvan, 2024). This project contains the following underlying data:
1. The raw file in Excel “LEMBAH PANTAI RAW DATA-1” 2. The tables in the Results section (Tables 3-10) “PPR Lembah Pantai TABLES” 3. The raw file in SPSS “SPSS LEMBAH PANTAI FINAL CLEAN” Data are available under the terms of the Creative Commons Zero “No rights reserved” data waiver (CC0 1.0 Public domain dedication). Dataverse: “Understanding the future of Dengue in Malaysia: Assessing Knowledge, Attitude, and Homeowner Practices in Mitigating Climate-Driven Risks”, https://doi.org/10.7910/DVN/9VNEET ( Galvan, 2024). This project contains the following extended data:
1. The questionnaire includes the full set of questions used to assess participants’ knowledge, attitudes, and practices related to dengue prevention in the Lembah Pantai area. Data are available under the terms of the Creative Commons Zero “No rights reserved” data waiver (CC0 1.0 Public domain dedication).
