# Supplementary material for: Understanding the future of dengue in Malaysia: Assessing knowledge, attitude, and homeowner practices in mitigating climate-driven risks
Source: F1000Res. 2024 Nov 12;13:1355. [Version 1] doi: 10.12688/f1000research.157226.1 (PMC12378623; doi:10.12688/f1000research.157226.1)
Supplement: Peer review history

# Reviewer report: Reviewer response for version 1

**DOI:** 10.5256/f1000research.172647.r397539

**Published:** 2025-08-25

**Authors:** Agung Sutriyawan

**Affiliations:** 1 Diponegoro University, Diponegoro, Indonesia; 2 Public Health, Bhakti Kencana University, Bandung, West Java, Indonesia

This study investigates the knowledge, attitudes, and practices (KAP) of residents in low-cost housing communities in Kuala Lumpur, Malaysia, concerning dengue fever and its prevention. Dengue fever remains a significant public health threat, and climate change is expected to exacerbate the spread of this disease due to changing weather patterns that support mosquito breeding.

 The study was conducted among 1,636 residents from four low-cost housing apartments, utilizing a structured questionnaire to assess their understanding and behaviors regarding dengue prevention. The results show that while many residents engage in preventive practices, the majority have poor knowledge about dengue and hold negative attitudes towards its prevention. The study identified several factors influencing KAP, including the PPR location, marital status, and prior dengue infection.

 The authors stress the need for community-based health education and tailored interventions that can bridge the knowledge gap, sustain preventive behaviors, and address the growing climate-driven risks associated with dengue.

Is the work clearly and accurately presented and does it cite the current literature?

Yes

If applicable, is the statistical analysis and its interpretation appropriate?

Yes

Are all the source data underlying the results available to ensure full reproducibility?

Yes

Is the study design appropriate and is the work technically sound?

Yes

Are the conclusions drawn adequately supported by the results?

Yes

Are sufficient details of methods and analysis provided to allow replication by others?

Yes

Reviewer Expertise:

Infectious diseases

I confirm that I have read this submission and believe that I have an appropriate level of expertise to confirm that it is of an acceptable scientific standard.

## References

[1] : Survey of knowledge and practices of dengue fever prevention related to sociodemographic status: community-based study in Bandung, Indonesia. *Journal of Public Health and Development* .2024;22(2) : 10.55131/jphd/2024/220203 26-38 10.55131/jphd/2024/220203

# Reviewer report: Reviewer response for version 1

**DOI:** 10.5256/f1000research.172647.r352160

**Published:** 2025-01-17

**Authors:** Juraina Abd-Jamil

**Affiliations:** 1 Universiti Malaya, Kuala Lumpur, Federal Territory of Kuala Lumpur, Malaysia

1. The paper is very well written and easy to read and understand. The authors have made extensive literature review and the study objectives very clear. Was there a specific reason that this study was conducted at only low-cost housing areas? Kindly include this in the manuscript.
2. The present study used structured questionnaires by Mac Guad et al (2021). Were terms used in the questionnaires adapted from the original questionnaires or changed to suit the needs of the present study? For example, terms such as “dengue carriers” – that ought to be referred to as “dengue vectors”.  Dengue carriers refer to the disease host carrying the virus. In addition, distinction between dengue virus and “dengue” the disease should be made clear in the survey as well. Consequently, were respondents assisted when answering the survey questions (to prevent any misunderstanding)? Kindly indicate in the manuscript what measures were taken to prevent or address any confusion or misunderstanding that might have masked true reflection of the respondent’s KAP on dengue.
3. Age stratification of respondents is not included in the manuscript. It would be interesting to see the respondents’ responses in the different age groups.
4. Categorisation of KAP is only divided to 2 groups, which are good or poor. Such categorisation is very limiting and does not reflect those individuals who had some knowledge on dengue, or some good attitude in preventing dengue. This group category should be captured considering that the study target group comes from lower socioeconomic background.

Is the work clearly and accurately presented and does it cite the current literature?

Yes

If applicable, is the statistical analysis and its interpretation appropriate?

Yes

Are all the source data underlying the results available to ensure full reproducibility?

Yes

Is the study design appropriate and is the work technically sound?

Yes

Are the conclusions drawn adequately supported by the results?

Yes

Are sufficient details of methods and analysis provided to allow replication by others?

Yes

Reviewer Expertise:

infectious diseases, vector-borne, dengue

I confirm that I have read this submission and believe that I have an appropriate level of expertise to confirm that it is of an acceptable scientific standard.
